# Marinobufagenin, Left Ventricular Hypertrophy and Residual Renal Function in Kidney Transplant Recipients

**DOI:** 10.3390/jcm12093072

**Published:** 2023-04-23

**Authors:** Davide Bolignano, Marta Greco, Pierangela Presta, Alfredo Caglioti, Nazareno Carullo, Mariateresa Zicarelli, Daniela Patrizia Foti, Francesco Dragone, Michele Andreucci, Giuseppe Coppolino

**Affiliations:** 1 Nephrology and Dialysis Unit, University “Magna-Graecia” of Catanzaro, 88100 Catanzaro, Italy; 2 Department of Medical and Surgical Sciences, University “Magna-Graecia” of Catanzaro, 88100 Catanzaro, Italy; 3 Clinical Pathology Lab, University “Magna-Graecia” of Catanzaro, 88100 Catanzaro, Italy; 4 Department of Health Sciences, University “Magna-Graecia” of Catanzaro, 88100 Catanzaro, Italy; 5 Department of Clinical and Experimental Medicine, University “Magna-Graecia” of Catanzaro, 88100 Catanzaro, Italy

**Keywords:** marinobufagenin, kidney transplantation, uremia, left ventricular mass index, left ventricular hypertrophy, biomarker

## Abstract

Background: Left ventricular hypertrophy (LVH), which is a pervasive complication of end-stage kidney disease (ESKD), persists in some uremic individuals even after kidney transplantation (Ktx), contributing to worsening CV outcomes. Marinobufagenin (MBG), an endogenous steroid cardiotonic hormone endowed with natriuretic and vasoconstrictive properties, is an acknowledged trigger of uremic cardiomyopathy. However, its clinical significance in the setting of Ktx remains undefined. Methods: In a cohort of chronic Ktx recipients (*n* = 40), we assessed circulating MBG together with a thorough clinical and echocardiographic examination. Forty matched haemodialysis (HD) patients and thirty healthy subjects served as controls for MBG measurements. Patients were then prospectively followed up to 12 months and the occurrence of an established cardio-renal endpoint (death, CV events, renal events, graft rejection) was recorded. Results: Median MBG plasma levels were lower in Ktx as compared with HD patients (*p* = 0.02), but higher as compared with healthy controls (*p* = 0.0005). Urinary sodium (β = 0.423; *p* = 0.01) and eGFR (β = −0.324; *p* = 0.02) were the sole independent predictors of MBG in this cohort, while a strong correlation with left ventricular mass index (LVMi), found in univariate analyses (R = 0.543; *p* = 0.0007), gained significance only in multivariate models not including eGFR. Logistic regression analyses indicated MBG as a significant predictor of the combined endpoint (OR 2.38 [1.10–5.12] per each 1 nmoL/L increase; *p* = 0.01), as well as eGFR, LVMi, serum phosphate and proteinuria. Conclusions: Ktx recipients display altered MBG levels which are influenced by sodium balance, renal impairment and the severity of LVH. Thus, MBG might represent an important missing link between reduced graft function and pathological cardiac remodelling and may hold important prognostic value for improving cardio-renal risk assessment.

## 1. Introduction

Kidney transplantation (Ktx) is the renal replacement therapy of choice for end-stage kidney disease (ESKD). Progressive renal recovery by the functional graft ameliorates various aspects of chronic uremia, such as blood pressure control, electrolytic imbalance, inflammation, oxidative stress and toxins overload, with a following improvement in the patients’ overall wellbeing and quality of life.

Yet, despite such tangible benefits, mortality remains remarkably higher in Ktx recipients as compared with the general population [1] and, in addition, cardiovascular (CV) disease ranks as the leading cause of death, more than infection or neoplasia [2].

Pathological left ventricular hypertrophy (LVH), which is largely prevalent among ESKD patients on chronic dialysis, persists in up to 50% of Ktx patients, representing an important prognostic factor for mortality, also among normotensive individuals [3]. LVH primarily arises as a maladaptive response to volume and pressure overload. However, persistence of this condition among Ktx recipients may recognize additional factors, including immunosuppressive therapy, genetic predisposition, hypoalbuminemia and, above all, residual uremic toxicity in less functional grafts.

Marinobufagenin (MBG) is a recently discovered endogenous cardiotonic steroid hormone, which binds the ubiquitous Na+/K+-ATPase cellular pump, triggering the activation of specific ionic intracellular signalling pathways [4]. Acute effects of MBG include natriuresis, peripheral vasoconstriction and the enhancement of cardiac inotropism. On the other hand, a chronic, sustained stimulation of the Na+/K+-ATPase activates diverse intracellular signalling pathways promoting collagen hyperproduction, cellular hypertrophy and deranged cell apoptosis [5], which underlies a causal involvement of MBG in pathological cardiac remodelling following various CV disorders, as well as in the pathogenesis of uremic cardiomyopathy (UCM) [6].

Circulating MBG levels are increased in chronic kidney disease (CKD) patients, indicating that an impaired renal function may somewhat alter the balance of this hormone [7]. Additionally, in ESKD patients undergoing chronic dialysis, higher MBG plasma levels parallel the severity of dysfunctional ventricular hypertrophy [8], an observation which is consistent with the alluded detrimental effects that a sustained exposure to MBG exerts on cardiac structure.

The possible role of this hormone in the setting of kidney transplantation, however, remains, thus far, undetermined.

Keeping this background in mind, we therefore aimed at running a pilot, observational, cross-sectional analysis to evaluate the clinical predictors of MBG in a small cohort of Ktx recipients, with a particular focus on a possible relationship with cardiac morpho-functional abnormalities and reduced graft function. In a following, exploratory prospective phase, we also tested the prognostic value of MBG as a risk biomarker for the occurrence of adverse cardio-renal events in the short–midterm.

## 2. Materials and Methods

### 2.1. Patients’ Selection

Fifty-eight adult kidney transplant recipients (age > 18 years) referred to the outpatient clinic of the University Hospital of Catanzaro, Italy, from November 2021 to February 2022 were screened to participate into this pilot, observational, prospective study. Infections, cancer, recent cardiovascular events requiring hospitalization, active inflammatory states, unstable renal function, severe renal impairment (GFR < 15 mL/min/1.73 m^2^, according to the CKD-Epi formula), evidence of chronic rejection, severe peripheral vasculopathy and recent transplantation (<3 months) represented the main exclusion criteria. The study was approved by the Local Institutional Review Board and all participating subjects provided written informed consent.

### 2.2. Clinical Assessment

A complete baseline assessment was performed in every participant upon hospital admission, before starting a planned outpatients visit. Clinical, demographic, and anthropometric parameters were recorded using a standardized, electronic case report form. Additionally, patients underwent blood pressure measurement at rest by a manual sphygmomanometer and a thorough echocardiographic examination. Left ventricular mass index (LVMi) was measured together with other cardiac morpho-functional parameters, as elsewhere recommended [9]. Blood specimens were collected in the morning after an 8 h overnight fast. Laboratory parameters were measured in all patients according to standard methods used in the clinical routine by a Cobas 8000 device (Roche Diagnostics, Basel, Switerland) and by an ADVIA 2120i machine (Siemens Healthcare Diagnostics, Marburg, Germany) using the relative kits. Serum samples were centrifuged at 1227× *g* for 15 min at 4 °C and the aliquots stored at −80 °C until thawed for batch analysis. MBG were measured in the blood using an ELISA commercially available kit (BlueGene Biotech, Shanghai, China), according to the manufacturer’s instructions. Measurements were made blind and in duplicate and levels were expressed as nmol/L. Circulating MBG levels were also quantified in a convenience sample of 30 healthy individuals and in 30 patients undergoing chronic renal replacement therapy by haemodialysis (HD) who were matched with Ktx recipients for age and gender.

### 2.3. Exploratory Follow-Up

After the baseline evaluation, patients entered an exploratory 12-month follow-up with an established cardio-renal endpoint of all-cause and CV mortality, non-fatal CV events (coronary, cerebrovascular or peripheral artery disease events, acute heart decompensation or severe cardiac arrythmia episodes requiring hospitalization), acute worsening in renal function (doubling of baseline serum creatinine or ≥25% decrease in baseline eGFR or ESKD requiring dialysis) or chronic graft rejection.

### 2.4. Statistical Analysis

The statistical analysis was performed using the SPSS package (version 24.0; IBM Corp., Armonk, NY, USA), the MedCalc Statistical Software (version 14.8.1; MedCalc Software bvba, MedCalc Software Ltd., Ostend, Belgium) and the GraphPad prism package (version 8.4.2, GraphPad Software, San Diego, CA, USA). Data were shown as mean ± SD, median (IQ range) or frequency percentage as appropriate. Differences between groups were assessed by the unpaired *t*-test for normally distributed values, the Mann–Whitney U test for non-parametric values and the chi-square followed by a Fisher’s exact test for frequency distributions. The Pearson (R) and the Spearman (Rho) correlation coefficients were employed to test correlations between variables, as appropriate. Univariate correlations were displayed graphically as linear regression. Before testing correlations, all values showing a skewed distribution were log-transformed to better approximate normal distributions. Multiple regression analyses were performed by building two separate models including all univariate correlates of MBG and LVMi values, respectively, in order to assess independent relationships. Data were expressed as partial correlation coefficients (β) and *p*-value. Univariate logistic regression analyses were performed to establish significant associations of clinical variables with the exploratory cardio-renal endpoint.

All results were considered significant for *p*-values ≤ 0.05.

## 3. Results

### 3.1. Main Characteristics of the Study Population

The final study population included 40 Ktx recipients. Mean age was 56.6 ± 12.5 years and 26 (65%) were male. Median transplantation vintage was 9 years (IQR 3–18), while the median dialysis duration before the transplant was 33.5 months (IQR 13–65). Most patients (85%) received a kidney from deceased donors. Only 10 patients (25%) were diabetics while the majority (65%) were hypertensive under pharmacological control, with 12 of them (30% of the whole population) being on RAAS blockers. Combined immunosuppressive therapy included calcineurin inhibitors in 37 patients (92.5%), corticosteroids in 33 (82.5%), mycophenolate mofetil in 29 (72.5%) and m-TOR inhibitors in only 4 patients (10%). Prevalence of other cardiovascular diseases was marginal. The median estimated GFR was 54.8 mL/min/m^2^ (IQR 32.7–66.1). All patients had no or negligible proteinuria (median 0.15 g/24 h; IQR 0.10–0.40). Serum calcium, phosphate and alkaline phosphatase levels were within the normal range in most individuals, as well as the lipid blood parameters and inflammatory profile. Echocardiography data indicated, on average, a preserved LV function (Ejection Fraction 59.3 ± 3.6%), mildly increased left atrial volume (35.1 ± 13 mL/m^2^) and minimal evidence of diastolic dysfunction (E/e’ 9.4 ± 3.9). LVMi was on average 104 ± 33 g/m^2^, with 15 individuals (37.5%) displaying pathologically increased values with respect to gender-adjusted cut-offs.

**Figure 1:**
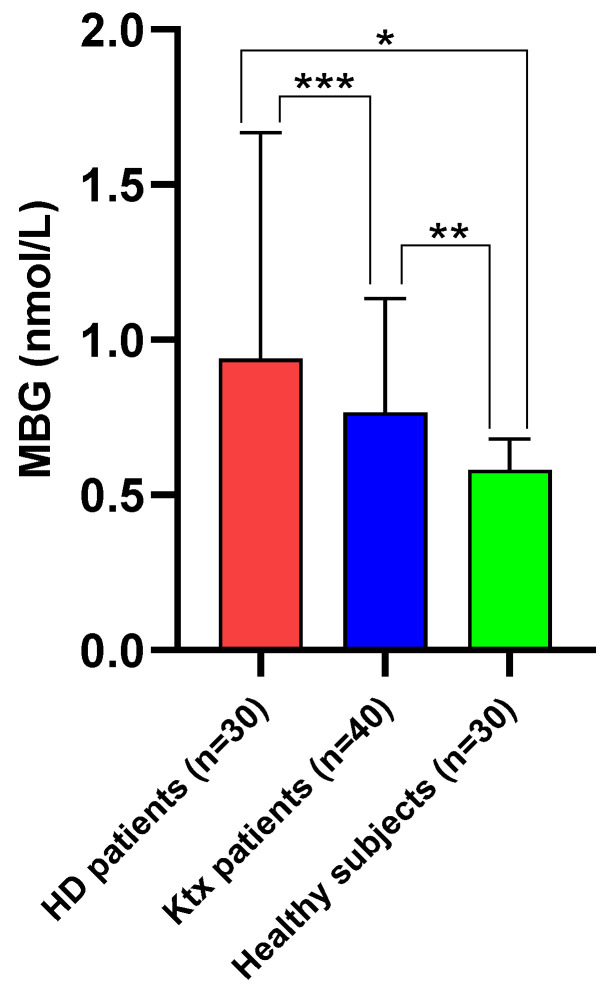
Median (IQR) circulating MBG levels in Ktx patients as compared with matched haemodialysis patients and healthy controls. * *p* < 0.0001; ** *p* = 0.0005; *** *p* = 0.02.

Median MBG plasma levels among Ktx were 0.765 [0.580–1.105] nmol/L. Such values were significantly higher as compared with those measured in healthy individuals (0.580 [0.240–0.660] nmol/L; *p* = 0.0005) but lower as compared with matched individuals on chronic HD treatment (0.940 [0.820–1.650] nmol/L; *p* = 0.02; Figure 1). Table 1 summarises the main anthropometric, clinical, laboratory and cardiovascular parameters of the study population.

### 3.2. Clinical Correlates of MBG

**Figure 2:**
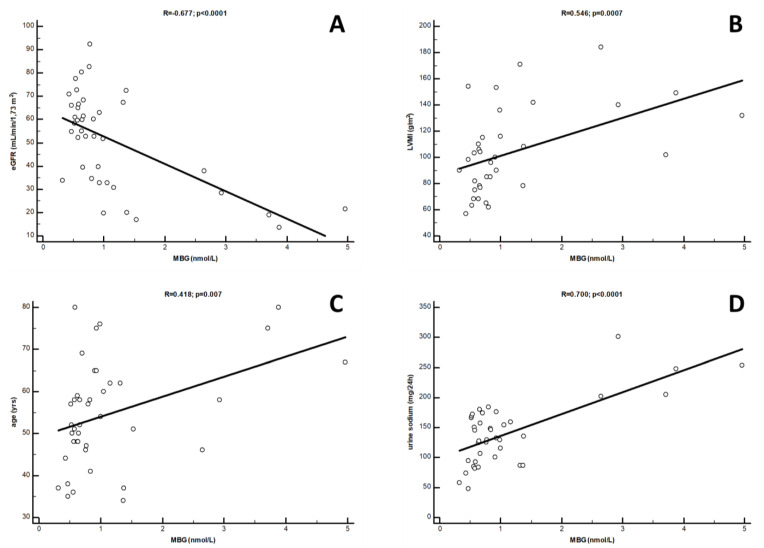
Univariate correlations between circulating MBG levels and (**A**) eGFR, (**B**) LVMi; (**C**) age and (**D**) urine sodium in Ktx patients.

From the univariate analysis, a strong inverse correlation was found between circulating MBG levels and eGFR values (R = −0.677; *p* < 0.0001). Conversely, robust direct correlations were found with urinary sodium excretion (R = 0.700; *p* < 0.0001), LVMi (R = 0.543; *p* = 0.0007) and age (R = 0.418; *p* = 0.007) (Figure 2), while a barely significant correlation was found with gender (Rho = 0.319; *p* = 0.04). In a multivariate model including all the univariate predictors of MBG, only the correlations with urinary sodium (β = 0.423; *p* = 0.01) and eGFR (β = −0.324; *p* = 0.02) were revealed as independent, while those with LVMi, age and gender were lost. This model was rather robust, explaining about 70% of the overall variation of MBG in this study cohort (*p* < 0.0001). Interestingly, LVMi re-emerged as a significant predictor of MBG after excluding eGFR from this model (β = 0.404; *p* = 0.02). Table 2 summarises data from uni- and multivariate analyses of MBG.

### 3.3. Clinical Correlates of LVMi

**Figure 3:**
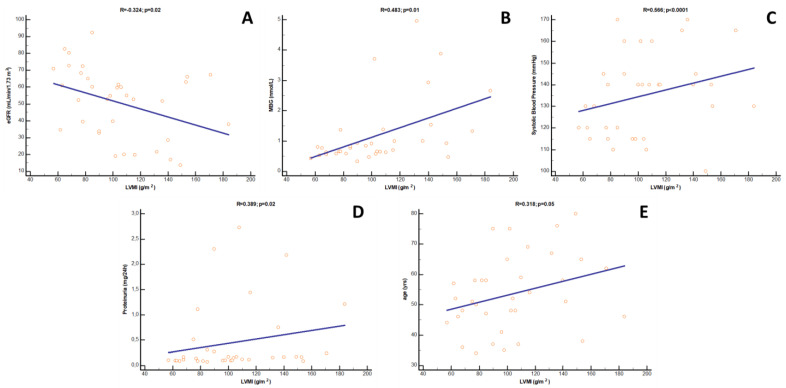
Univariate correlations between LVMi and (**A**) eGFR, (**B**) MBG, (**C**) systolic blood pressure, (**D**) proteinuria and (**E**) age in Ktx patients.

At univariate analysis, LVMi was directly correlated with systolic BP (R = 0.566; *p* < 0.0001), MBG (R = 0.483; *p* = 0.01), proteinuria (R = 0.389; *p* = 0.02) and, marginally, with age (R = 0.318; *p* = 0.05), while an inverse correlation was found with eGFR (R = −0.374; *p* = 0.02) (Figure 3). As revealed by multivariate analysis, however, only the relationships with systolic BP (β = 0.319; *p* = 0.01) and eGFR (β = −0.201; *p* = 0.01) were maintained as independent, while those with proteinuria and age disappeared. Of note, in this fully adjusted model, the correlation with MBG remained statistically borderline (β = 0.257; *p* = 0.08), attaining overt significance only if eGFR was excluded from the model (β = 0.317; *p* = 0.001). Likewise, in this latter case, also the association with age re-appeared (β = 0.201; *p* = 0.04). Results from uni- and multivariate analyses of LVMi are depicted in Table 3.

### 3.4. Exploratory Cardio-Renal Endpoint in Ktx Recipients

**Figure 4:**
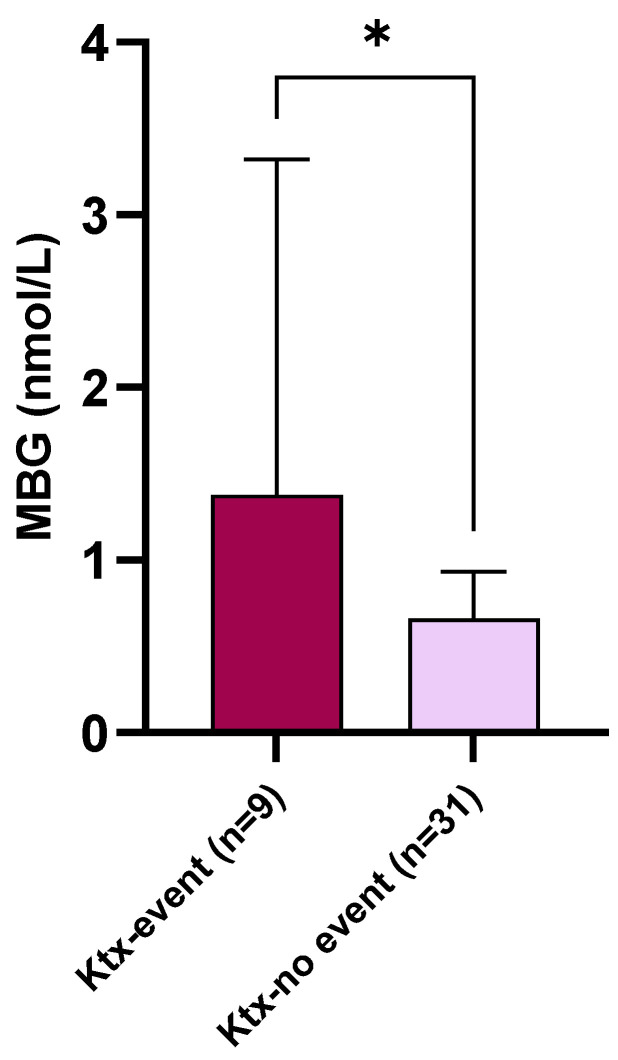
Baseline circulating MBG levels in Ktx patients experiencing the combined cardio-renal endpoint as compared with others. * *p* = 0.02.

During the follow-up (median 10 mo.; range 2–12), nine Ktx patients (22.5%) reached the composite cardio-renal endpoint. In further detail, two patients died, two patients had a non-fatal CV event, while the remaining five had a renal event. Baseline MBG plasma levels were significantly higher in Ktx individuals experiencing the endpoint as compared with others (1.38 [0.84–3.12] vs. 0.66 [0.57–0.92] nmol/L; *p* = 0.02. Figure 4). At univariate logistic regression analysis (Table 4), significant predictors of the study endpoint were eGFR (OR 0.90; [95% CI 0.84–0.96] per each 1 mL/min/1.73 m^2^ increase; *p* < 0.0001), serum phosphate levels (OR 3.23 [1.46–5.76] per each 1 mg/dL increase; *p* = 0.0001), LVMi (OR 1.04 [1.01–1.06] per each 1 g/m^2^ increase; *p* = 0.0003, proteinuria (OR 3.37 [1.51–6.04] per each 100 mg/24 h increase; *p* = 0.002) and MBG plasma levels (OR 2.38 [1.10–5.12] per each 1 nmoL/L increase; *p* = 0.01).

## 4. Discussion

In the present study, we have investigated, for the first time, the possible clinical significance of circulating MBG in a homogeneous cohort of Ktx recipients. Although preliminary, various findings from our study deserve, in our opinion, a focussed discussion.

First, circulating MBG levels in Ktx recipients were, on average, lower as compared with chronic haemodialysis patients but higher with respect to healthy individuals.

Under physiological conditions, MBG is produced and released from adrenocortical glands in response to high plasma sodium, volume overload and angiotensin-2 increase, despite additional, yet unknown mechanisms having been postulated. Acute peripheral effects of MBG include vasoconstriction and natriuresis. MBG is freely filtered in urine but the excretion mechanism, overall clearance and peripheral metabolism still remain largely undefined [4].

A sustained MBG accumulation in uremic patients is a well-acknowledged issue and may portend serious detrimental effects in the long term. In fact, a persistent activation of the Na+/K+-ATPase elicits pathological vascular and cardiac remodelling and aberrant fibrosis, thereby causing or worsening existing CV damage [10]. For these reasons, MBG is nowadays considered a true uremic toxin, as well as one the most important known triggers of uremic cardiomyopathy [11]. Functional recovery by the kidney graft may theoretically normalize circulating MBG balance. However, Ktx individuals in this cohort displayed on average a mild-to-moderately impaired renal function, which is comparable to the presence of a chronic kidney disease (CKD) stage 3–4. Hence, predictably, MBG plasma levels in Ktx remained somewhat higher as compared with those measured in a group of healthy matched controls. A crucial impact of residual renal function on MBG balance is further supported by the strong (inverse) correlation found in this cohort between MBG and eGFR values. Of note, this association remained robust and fully independent from potential confounders even after multivariate adjustment, a finding which pairs well with previous observations in non-advanced CKD patients [7]. At present, the mechanism through which a reduced renal function may trigger an increase in MBG plasma levels remains unclear. An impaired clearance by the kidney itself or a reduced peripheral catabolism cannot, in principle, be ruled out, but mechanistic evidence in this regard is lacking. Additionally, an hyperactivation of the renin-angiotensin system and a chronic volume overload—two key hallmarks of CKD—are powerful inductors of MBG synthesis and could therefore be called into question [12]. Finally, and no less importantly, given the steroid nature of MBG, a possible influence of chronic steroid immunosuppressive therapy cannot in principle be ruled out. Nevertheless, under either physiological or pathological conditions, chronic sodium overload remains the principal stimulus to MBG release, in line with the natriuretic properties of this substance [13]. Accordingly, in our study cohort, urinary sodium (which largely reflects daily sodium intake) ranked as the strongest predictor of circulating MBG. Again, such a strong association was confirmed as independent at multivariate analysis; this indicates that, even in such a particular population setting, an excessive sodium consumption remains one of the most significant triggers of MBG.

Another important aim of our study was to investigate possible relationships between MBG and cardiac remodelling in Ktx recipients. As alluded to before, a plethora of observations pointed at MBG as an important factor eliciting myocardial hypertrophy and, lately, fibrosis in experimental models of uremia [6]. Likewise, direct relationships between circulating MBG and abnormal cardiac remodelling in dialysis patients have already been described [8,14].

In renal patients, the prevalence of left ventricular hypertrophy parallels the severity of renal function impairment, resulting in affecting roughly 70–80% of uremic patients on chronic dialysis [15].

Although the recovery in renal function driven by Ktx may ameliorate cardiac remodelling, LVMi remains increased in some Ktx recipients [3]. Indeed, in our cohort, a substantial percentage of Ktx recipients displayed evidence of a mildly augmented LVMi and, importantly, the results showed eGFR as the strongest predictor of ventricular mass among all the clinical factors considered. On the other hand, we have reported a strong direct association between MBG and LVMi, but such an association was apparently not independent, as emerged in the two different, fully adjusted multivariate models. Of note, in both models, the association between MBG and LVMi regained significance after excluding eGFR from the adjustment. Hence, taken altogether, these observations might place MBG in the complex pathogenetic chain linking renal function impairment to the severity of left ventricular hypertrophy, which further supports the role of this substance as an important cardiotoxic uremic toxin.

In previous studies, MBG has proven to be useful as a prognostic biomarker in predicting mortality among HD patients [16], CV events in individuals with chronic heart failure [17] and even a more accelerated eGFR decline among individuals with mild CKD due to arterial hypertension [7].

Hence, apart from its involvement in the pathophysiology of cardio-renal damage, MBG is now attracting increasing attention as a potential additive tool for risk stratification.

Accordingly, an exploratory logistic regression analysis made in our Ktx recipients revealed a significant association between MBG plasma levels and the occurrence of a combined cardio-renal endpoint within a 12-month follow-up. Of note, such an association was evident despite the very low number of participants and the few events recorded. Unfortunately, the study was not sufficiently powered to answer this particular research question in a proper manner, either in terms of population size or follow-up length: this prevented us to perform more complex survival analyses to verify whether the prognostic value of MBG would have been fully independent from other acknowledged risk predictors such as, for instance, eGFR and LVMi.

## 5. Conclusions

In this pilot study, we have described for the first time altered MBG levels in Ktx recipients which are strictly influenced by sodium balance and, above all, by the entity of renal function impairment and the severity of left ventricular hypertrophy. MBG might thus be placed at the crossroad of pathological cardiac remodelling and residual graft function, although its biological significance as a causal factor or a simple epiphenomenon cannot be clarified in the absence of proper mechanistic evidence. In this latter regard, future prospective investigations on larger and more heterogeneous cohorts are desirable to confirm the prognostic usefulness of this substance for improving cardio-renal risk assessment, thus extending to Ktx recipients the promising observations made in other disease settings.

## Figures and Tables

**Table 1:** Main anthropometric, clinical and laboratory parameters of the study population.

*n* = 40	
Age (yrs)	56.6 ± 12.5
Gender (%Male)	65
Dialysis vintage (mo.)	33.5 [13–65]
Ktx vintage (yrs)	9 [3–18]
DD Ktx (%)	85
BMI (kg/m^2^)	26.1 ± 4.9
WHR (cm/cm)	0.92 ± 0.08
Current smokers (%)	15
Immunosuppressive Therapy (%):	
- Corticosteroids	82.5
- CNI	92.5
- MMF	72.5
-m-TORi	10
Any RAAS blocker (%)	30
Diabetes (%)	25
Coronary disease (%)	2.5
Heart failure (%)	5
Hypertension (%)	65
SBP (mmHg)	135 ± 18
DBP (mmHg)	85 ± 6.6
LAVi (mL/m^2^)	35.1 ± 13
LVMi (g/m^2^)	104 ± 33
Ejection Fraction (%)	59.3 ± 3.6
Vmax (m/s)	2.06 ± 0.54
TAPSE (mm)	21.8 ± 2.2
E/e’	9.4 ± 3.9
RAVi (mL/m^2^)	18.3 ± 7.3
Glycemia (mg/dL)	96.5 ± 22.9
eGFR (CKD-EpimL/min/1.73 m^2^)	54.8 [32.7–66.1]
Proteinuria (g/24 h)	0.15 [0.10–0.40]
Urine sodium (mg/24 h)	142.2 ± 52.2
Urine potassium (mg/24 h)	48 [41.2–60]
Urea (mg/dL)	55 [43.5–89.5]
Serum Phosphate (mg/dL)	3.34 ± 0.97
Serum Calcium (mg/dL)	9.6 ± 0.82
Parathormone (pg/mL)	122.7 [68.6–167.2]
Alkaline Phosphatase (U/L)	80.4 ± 20.5
Total Cholesterol (mg/dL)	184.6 ± 36.7
LDL Cholesterol (mg/dL)	109.5 ± 34.2
Triglycerides (mg/dL)	140.5 ± 55.8
Fibrinogen (mg/dL)	352.3 ± 99.1
ESR (mm/h)	15 [9–27]
Albumin (g/dL)	4.38 ± 0.35
RBC (*n* × 10^6^)	4.68 ± 0.82
Hb (g/dL)	12.6 ± 1.9
C-reactive protein (mg/L)	3.23 [2.13–4.10]
Ferritin (mg/dL)	38 [16.5–97]
TSAT (%)	31.7 ± 5.7
Serumiron (mg/dL)	66.1 ± 28.9
MBG (nmol/L)	0.765 [0.580–1.105]

BMI, body mass index; DBP, diastolic blood pressure; DD, deceased donor; E/e’, early diastolic peak left ventricular inflow velocity (E)/early diastolic peak lateral mitral annular velocity (e’) ratio; eGFR, estimated glomerular filtration rate; ESR, erythrocyte sedimentation rate; Hb, haemoglobin; Ktx, kidney transplantation; LAVi, left atrial volume index; LDL, low density lipoprotein; LVMi, left ventricular mass index; MBG, marinobufagenin; RAVi, right atrial volume index; RBC, red blood cells; SBP, systolic blood pressure; TAPSE, tricuspid annular plane excursion; TSAT, saturated transferrin; Vmax, peak aortic valve velocity; WHR, waist–hip ratio.

**Table 2:** Univariate and multiple regression analysis of (log)MBG levels.

	Univariate Correlation Coefficient	*p*
(log)eGFR	−0.677	<0.0001
Urinary Sodium	0.700	<0.0001
LVMi	0.546	0.0007
Age	0.418	0.007
Gender	0.319	0.04
	**Multivariate Standardized Correlation Coefficient (β)**	***p***
Urinary Sodium	**0.423**	**0.01**
(log)eGFR	**−0.324**	**0.02**
LVMi	0.244	0.11
Age	0.756	0.45
Gender	0.594	0.55

Multiple R = 0.84, R^2^ = 70%; *p* < 0.0001. Statistically significant correlations at multivariate analyses are highlighted in bold.

**Table 3:** Univariate and multiple regression analysis of LVMi values.

	Univariate Correlation Coefficient	*p*
Systolic Blood Pressure	0.566	<0.0001
(log)MBG	0.483	0.01
(log)eGFR	−0.374	0.02
(log)Proteinuria	0.389	0.02
Age	0.318	0.05
	**Multivariate Standardized Correlation Coefficient (β)**	***p***
Systolic Blood Pressure	**0.319**	**0.01**
(log)eGFR	**−0.201**	**0.01**
(log)MBG	0.257	0.08
(log)Proteinuria	0.231	0.25
Age	0.181	0.32

Multiple R = 0.61, R^2^ = 37%; *p* = 0.006. Statistically significant correlations at multivariate analyses are highlighted in bold.

**Table 4:** Clinical variables significantly associated with the combined cardio-renal endpoint at univariate logistic regression analysis.

	Unit of Increase	OR	95% CI	*p*
eGFR	1 mL/min/1.73 m^2^	0.90	0.84–0.96	<0.0001
Serum Phosphate	1 mg/dL	3.23	1.46–5.76	0.0001
LVMi	1 g/m^2^	1.04	1.01–1.06	0.0003
Proteinuria	100 mg/24 h	3.37	1.51–6.04	0.002
MBG	1 nmoL/L	2.38	1.10–5.12	0.01

eGFR: estimated glomerular filtration rate; LVMi: left ventricular mass index; MBG: marinobufagenin.

## Data Availability

Raw data from the present study are available from the Corresponding Author upon reasonable request.
